# Development of Whole-Genome Agarose-Resolvable LInDel Markers in Rice

**DOI:** 10.1186/s12284-019-0361-3

**Published:** 2020-01-06

**Authors:** Wei Hu, Tianhao Zhou, Pengfei Wang, Bo Wang, Jiaming Song, Zhongmin Han, Lingling Chen, Kede Liu, Yongzhong Xing

**Affiliations:** 1 National Key Laboratory of Crop Genetic Improvement, Huazhong Agricultural University, Wuhan, 430070 China; 2 College of Agriculture, Yangtze University, Jingzhou, 434000 China

**Keywords:** Large fragment insertion/deletion, Agarose-resolvable LInDel marker, Polymorphism information contents, Heteroduplex, Breeding markers

## Abstract

The level of difficulty involved in separating marker genotypes greatly determines the utilization of such marker-aided selection (MAS) by breeders. Genotyping by use of agarose gel electrophoresis is easily accepted by breeders due to its simple requirements and easy operation in the lab. Here, we extracted 19,937 large fragment insertions/deletions (LInDels) that were 30–55 bp based on two *indica* rice and one *japonica* rice reference genome sequences. Thousands of primer pairs were designed by the Primer 3 program to amplify the corresponding LInDels, and 6582 LInDel markers with unique genome loci were reserved after being tested by e-PCR; 346 of these markers were validated in a panel of 22 cultivars by running on a 1.5% agarose gel. Most LInDel markers had a considerable number of polymorphisms. The LInDel markers have an equivalent efficiency to that of the SSR and SNP markers in identifying hybrids, estimating genetic distance and developing genetic linkage maps. The hybrid genotypes of the LInDel markers exhibited three bands, which were the result of heteroduplex formation between the insertion allele and the deletion allele. Fifty-five breeding markers, including 9 intragenic markers and 46 closely linked LInDel markers, were developed for 55 known genes that are related to yield, biotic and abiotic stress tolerance. These agarose-resolvable LInDel markers will be welcomed by breeders and will play an important role in MAS.

## Introduction

DNA markers have been widely used for genetic improvement of rice due to their abundant number and even distribution across the genome. Since the 1980s, three generations of DNA markers have been developed and widely utilized for the genetic improvement of plants. The first generation marker is restriction fragment length polymorphism (RFLP); RFLPs are detected by restriction enzyme digestion and hybridization with isotope-labeled or biotin-labeled probes (McCouch et al. 1988; Williams et al. 1991). The second generation marker is detected by polymerase chain reaction (PCR) such as random amplified polymorphic DNAs (RAPD) (Fukuoka et al. 1992; Williams et al. 1990) and simple sequence repeats (SSR) (Tautz 1989; Temnykh et al. 2000), which are mainly separated by polyacrylamide gel electrophoresis. The third generation DNA markers are single nucleotide polymorphism (SNP) markers and insertion/deletion (InDel) markers, which are detected by sequencing (Feltus et al. 2004). The first-generation markers have seldom been utilized in plant genetics in recent years due to their time-consuming detection and the harm they cause to the environment. The polymorphism of SSR markers is caused by a variable number of 2 to 6 bp tandem repeats, most of which need to be separated by polyacrylamide gel electrophoresis. SSR markers are popular due to their characteristics of co-dominance, multiallelism, easy reproducibility, and their random and wide distribution (Rallo et al. 2000); these characteristics have made great contributions to the development of genetic linkage maps and MAS in rice (McCouch et al. 1997; McCouch et al. 1988). For SNPs genotyping, PCR agarose gel-based genotyping are available such as tetra primer PCR method (Kim et al. 2016) and cleaved amplified polymorphic sequence (CAPS) markers (Fan et al. 2009). With the development of next-generation sequencing, SNPs and InDel have been more popular in plant studies due to their co-dominant, genome-wide distribution and low cost. However, genotyping SNP markers depends on sequencing or on SNP arrays, which are not evaluated in most breeding units. Therefore, SNP genotyping has been frequently performed by commercial companies, and it takes a great deal of time to achieve the data. InDel markers are also separated by running an agarose or polyacrylamide gel, depending on the size of the InDel. The large fragment insertions/deletions (LInDel) markers are designed to amplify 300–350 bp fragments, which cover the variations of large insertions/deletions of 30–55 bp and are easily separated by agarose gel electrophoresis due to a 10%-fragment length polymorphism. For most breeders, agarose gel electrophoresis equipment is easy to obtain; thus, LInDel markers are expected to be used for quickly genotyping the plants that are produced in the breeding program. This makes the breeding process more autonomous and enhances the work efficiency. Therefore, a large number of genome-wide LInDel makers are urgently needed. With the release of the genome reference sequence, InDel markers have been confirmed to be evenly spread throughout the whole genomes of many crop plants, such as cotton, rice, maize, oilseed rape and so on (Li et al. 2014; Liu et al. 2015a; Liu et al. 2015b; Mahmood et al. 2016; Moghaddam et al. 2014; Shen et al. 2004; Song et al. 2015).

Currently, two sets of rice InDel markers have been designed and released for public use. Shen et al. extracted SNPs and InDels between Nipponbare and 9311 genome sequences and proved that 90% of the InDel polymorphisms could be developed into InDel markers. They designed primers to amplify 120 to 250 bp fragments covering InDels of 25–50 bp. Only 108 pairs of InDel primers were designed and verified through experimental validation (Shen et al. 2004). Besides, these markers were validated by running 3.5% agarose gel electrophoresis, which would take longer running time to separate the InDel differences. Liu et al. designed InDel markers for both polyacrylamide gel electrophoresis and agarose gel electrophoresis based on the InDels extracted from 1767 rice genomes (Liu et al. 2015a). For the InDel sizes ranging from 2 to 7 bp, primers were designed to amplify the PCR products of 60–100 bp, which were separated by polyacrylamide gel electrophoresis. For InDels sizes larger than 7 bp, primers were designed to amplify PCR products of 150–300 bp, which were separated by agarose gel electrophoresis. The average size of their PCR products was 220 bp for an average InDel size of 15 bp, and this might be difficult to resolve with an agarose gel. Thus, genome-wide, LInDel markers are needed for convenient use in rice genetic improvement.

The high-quality genome reference sequences of two Chinese elite *indica* rice varieties, Zhenshan 97 (ZS97) and Minghui 63 (MH63), were recently released to public (Zhang et al. 2016a). When comparing the references sequences, the following InDels were found: 0.70 InDel per kilobase for the ZS97 and MH63 reference sequences; 1.31 InDels per kilobase for the reference sequences of ZS97 and Nipponbare; and 1.33 InDels per kilobase for the MH63 and Nipponbare reference sequences (Zhang et al. 2016b). Such high-density InDels provide the chance to screen for valuable LInDel markers in *indica* intra-subspecies and inter-subspecies, which can distinguish the genotype identity via agarose gel electrophoresis rather than polyacrylamide gel electrophoresis. In this study, we screened 1838 LInDels (insertions/deletions ranging from 30 bp to 55 bp) between the ZS97 and MH63 genome sequences and 4744 LInDels between the two *indica* genome sequences and the Nipponbare genome sequence. To validate the quality of these LInDel markers, 310 markers derived from the InDels between ZS97 and MH63, which were approximately evenly distributed on 12 chromosomes, were chosen to test a panel of 22 rice varieties, including the *indica* and *japonica* varieties. In addition, 36 LInDel markers derived from the InDels between ZS97 and Nipponbare or between MH63 and Nipponbare were also validated. The genetic distance of the 22 varieties was calculated based on the genotypes at these verified marker loci. In addition, 20 LInDel markers on chromosome 1 were selected to develop a genetic linkage map using the F_2_ population derived from ZS97 and MH63. In addition, 55 breeding markers, located in the intragenic regions of important genes or closely linked to important genes, were designed or selected for MAS. Thus, these LInDel markers provide convenience for rice genetic studies and MAS.

## Materials and Methods

### Plant Materials

The genomic DNA of 22 rice varieties, including 7 *indica* varieties (ZS97, MH63, N22, 9311, Teqing, Heimichut and Malaixizidao), 11 *japonica* varieties (Zhonghua 11, Xizang 2, Nipponbare, 02428, Taichung 65, Haobuka, Wuyugeng, Zidao, Xiushui 134, Mitak and Cypress), 2 *Aus* cultivars (Chuan7, AUS449) and two unclassified cultivars (H360 and Gangye) were isolated from fresh leaves. The classification of the cultivars was based on the RiceVarMap database (http://ricevarmap.ncpgr.cn/v2/). A total of 96 F_2_ plants from the hybrid between ZS97 and MH63 was used to develop the genetic linkage map. The genomic DNA was isolated with a modified CTAB method (Murray and Thompson 1980).

### LInDel Marker Screening

The InDels between ZS97 and MH63 were extracted using the MUMmer (version 3.23) software (Delcher et al. 2002). The specific parameters were specified following Zhang’s method (Zhang et al. 2016b). The genome sequences (the first release version) of ZS97 and MH63 were downloaded from the Rice Information Gateway database (http://rice.hzau.edu.cn/cgi-bin/rice/download_ext). The InDels between ZS97 and Nipponbare or between MH63 and Nipponbare were extracted using the same method. The Nipponbare genome reference was downloaded from the Rice Genome Annotation Project website (http://rice.plantbiology.msu.edu/pub/data/Eukaryotic_Projects/o_sativa/annotation_dbs/pseudomolecules/version_7.0/). All InDel markers with insertions/deletions of 30 to 55 bp (LInDel) were collected as candidate markers.

### Design of LInDel Markers

The design of the primers to amplify the LInDel markers must meet the following two conditions: (1) The primers for the LInDel markers must be strictly unique and should not be mismatched to other loci. (2) The PCR products of the LInDel markers should be 300–350 bp so that the polymorphism among the genotypes can be easily identified with the naked eye on a 1.5–2% agarose gel. The LInDel markers were designed using Primer 3 (Rozen and Skaletsky 2000). The specific parameters were set as follows: the primer length was set at 20 to 24 bp with an optimal length of 22 bp, the melting temperature (Tm) was set at 58 to 63 °C with an optimal temperature of 60 °C, and the GC content of the primer ranged from 40% to 60% with an optimal GC content of 50%. Before the final markers were confirmed, an e-PCR program was run to guarantee the uniqueness of the designed markers (Schuler 1997). The ZS97, MH63 and Nipponbare genome sequences were set as the references for the e-PCR program. Only the markers that matched unique loci among the three reference genomes were utilized. All the processes were carried out using custom Perl scripts.

### Validation of the InDel Markers

A total of 346 LInDel markers (including 310 LInDel markers from ZS97 and MH63 and 36 LInDel markers from Nipponbare and ZS97 or MH63) were chosen from the markers designed with the program. To examine the versatility of these markers, 22 varieties were genotyped with these markers. The PCR program was set as follows: approximately 40–60 ng of DNA template, 1.5 μL of 10x buffer (Mg^2+^ plus), 0.75 μL of dNTP (2 mM), 2.5 nM of each primer, 0.5 U of rTaq polymerase (Takara Bio, Beijing), and ddH_2_O was added to a final reaction volume of 15 μL. The reaction mixture was initially denatured at 94 °C for 3 min; followed by 35 cycles of amplification at 94 °C for 30 s, 58 °C for 30 s and 72 °C for 30 s; and a final extension at 25 °C for 1 min. PCR was performed using a 2720 Thermal Cycler (Applied Biosystems, USA). The PCR products were separated by running a 1.5% (w/v) agarose gel mixed with 3% GoldView Dye (Zomanbio, Beijing, China).

### Calculation of the Polymorphism Information Content of the LInDel Markers

Twenty-two varieties were genotyped with these 346 LInDel markers by separating the PCR products with an agarose gel. Each band represented one genotype and defined a special Arabic numeral. Finally, all the different band types were counted based on their Arabic numerals, and the frequency of each type was calculated. The polymorphism information content (PIC) was calculated following the equation: ${\mathrm{PIC}}_i=1-{\sum}_{j=1}^n{p^2}_{ij}$, where *p*_*ij*_ was the frequency of the *j*th type for the *i*th marker (Anderson et al. 1993; Liu et al. 2015a).

### Cluster Analysis

A total of 312 polymorphic markers, including 279 validated markers from the ZS97/MH63 LInDels and 33 validated markers from the ZS97/Nipponbare or the MH63/Nipponbare LInDels, were used for cluster analysis. The genetic distance was calculated by NTSYS 2.1 (Rohlf 2002), and then the MEGA X (Kumar et al. 2018) was used for UPGMA cluster analysis based on the genetic distance calculated.

### Developing a Linkage Map with LInDel Markers

Twenty markers on chromosome 1 were used to genotype the 96 F_2_ individuals. The genetic linkage map was constructed using Mapmaker 3.0 (Lincoln et al. 1993). The Kosambi function was used to convert the recombination values to the genetic distances in centimorgans (cM) (Kosambi 1944).

### Design of the LInDel Markers Related to Important Genes

To develop intragenic LInDel markers for important genes, 55 agronomically important genes were targeted from the China Rice Data Center (http://www.ricedata.cn/gene/). These genes included the following: 15 rice blast resistance genes; 8 bacterial blight resistance genes; 5 brown planthopper resistance genes; 4 heading date genes; 8 grain size/shape genes and 15 other genes, including yield-related genes, cold or heat resistance genes and plant architecture genes. More details on these genes are shown in Additional file 9. The alleles of ZS97, MH63 and Nipponbare for these 55 genes were downloaded from the Rice Information Gateway database(http://rice.hzau.edu.cn/rice_rs1/) and the Rice Genome Annotation Project database (http://rice.plantbiology.msu.edu/). For all these genes, no alleles contained LInDels of 30–55 bp among the three varieties. Therefore, we included the InDels of the 16–30 bp fragment for marker development. If no appropriate InDel markers were presented within the gene sequence, the most closely linked LInDel marker (a distance less than 200 kb between the marker and gene) was chose as an alternative (Additional file 9).

## Results

### Identification of the LInDels between ZS97 and MH63

A total of 251,837 InDels were discovered between the ZS97 and MH63 reference genomes with an average density of 0.70 InDel/kb (Zhang et al. 2016b). Among them, 10,361 LInDels had large insertion/deletion sizes ranging from 20 bp to 90 bp, which could be separated by agarose gel electrophoresis. More than half of the LInDels fell into the size range of 20–29 bp. Most chromosomes contained 600 to 900 LInDels, while chromosomes 1, 3 and 11 contained more than one thousand. Chromosome 7 contained the smallest number of LInDels at 542 (Additional file 1: Figure S1). The number of LInDels decreased as the InDel size increased (Additional file 1: Figure S2; Additional file 2). The average number of LInDels varied from 23.5 per kilobase (on chromosome 11) to 50.5 per kilobase (on chromosome 7) (data not shown).

### Design of Agarose-Resolvable LInDel Markers between ZS97 and MH63

**Fig. 1:**
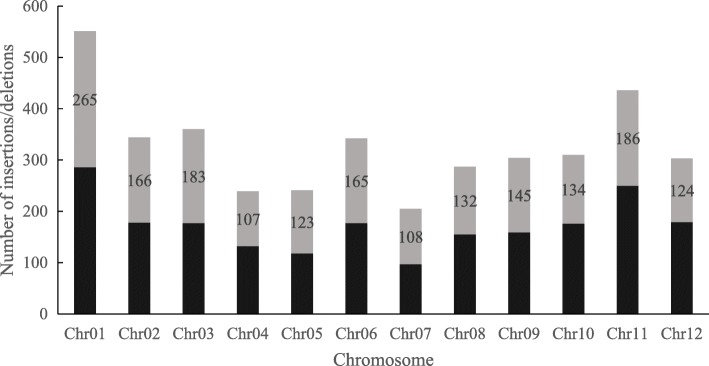
The total number of 30–55 bp insertions/deletions and the number of potential agarose-resolvable LInDel markers. The total bar represents the total number of LInDels on each chromosome; the number in the gray bar represents the number of unique agarose-resolvable LInDels markers

With the InDel genomic distribution and easy separation by agarose gel electrophoresis, 30–55 bp InDels were targeted for the development of LInDel markers. One kilobase fragments from ZS97 and MH63 sequences that harbored the corresponding LInDels were extracted as templates for primer design. The fragments that amplified an amplicon of 300 to 350 bp with the designed primers were utilized for marker development. Therefore, 3922 primer pairs were designed to amplify their corresponding LInDels for marker development (Additional file 3). These 30–55 bp LInDels shared a genome-wide distribution similar to the distribution of 20–90 bp LInDels (Fig. 1 and Additional file 1: Figure S1). No available primers were designed for the 362 candidate fragments in either ZS97 or MH63. Available primers for the 644 candidate fragments were designed only in either the ZS97 reference sequence or the MH63 reference sequence. In addition, the primers designed based on the 1078 candidate fragments were excluded by an e-PCR program test that revealed multiple matching copies with the primer pairs in the genome. Finally, 1838 primer pairs were successfully designed to uniquely amplify the corresponding LInDels (Additional file 4). There were at least 100 LInDels on each chromosome. In general, nearly half of the LInDels could be designed as agarose-resolvable LInDel markers (Fig. 1).

### Design of Agarose-Resolvable LInDel Markers between the *indica* and *japonica* Rice Species

Following the strategy conducted in *indica* intra-subspecies rice, we extracted LInDels between ZS97 and Nipponbare and between MH63 and Nipponbare. The number of obtained LInDels with insertion/deletion sizes of 30–55 bp was 7923 and 8092, respectively (Additional file 5). Finally, 3535 and 3619 unique primer pairs were successfully designed for the LInDel amplicons through an e-PCR test. A total of 2410 primer pairs were common between the two crosses. Finally, 4744 unique LInDel markers were developed (Additional file 6).

A comparison of the three sets of unique LInDel markers (including 1838 markers from ZS97/MH63, 3535 markers from ZS97/Nipponbare and 3619 markers from MH63/Nipponbare) showed that 509 markers were valuable both in ZS97/MH63 and MH63/Nipponbare, and 566 markers were valuable in both ZS97/MH63 and ZS97/Nipponbare. A total of 2410 markers were valuable for both the MH63/Nipponbare and ZS97/Nipponbare InDels (Additional file 1: Figure S3). As we expected, no LInDel markers showed polymorphism between any two genotypes.

### Validation of the LInDel Markers

**Fig. 2:**
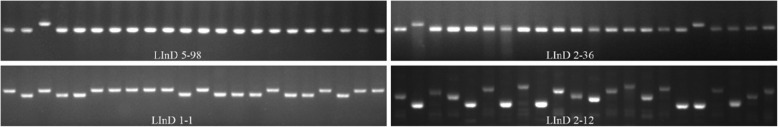
The genotype of four representative LInDel markers of LInD5–98, LInD2–36, LInD1–1 and LInD2–77. The samples from left to right are as follows: Zhonghua11, Zhenhan97, Minghui63, Xizang2, N22, Nipponbare, 9311, 02428, Teqing, Taichong65, Chun7, Haobuka, Wuyujing3, Gangye, H360, Zidao, Heimizhan, Changguizidao, Xiushui133, AUS449, Mitak and Cypress

To check the availability of these potential LInDel markers, 310 primer pairs that were designed based on the LInDels between ZS97 and MH63 were used to amply the InDels from 22 varieties (Additional file 7). Agarose gel electrophoresis of the PCR products showed that the 9 primer pairs could not amplify any PCR products from the 22 rice varieties, and 10 primer pairs amplified uniform length amplicons among all 22 varieties. In addition, 12 pairs amplified nonspecific products. In conclusion, 289 of the total primers could amplify clear PCR products, while 279 of them exhibited polymorphisms among the 22 varieties (Additional file 8). A total of 97.1% (301/310) of the primers amplified specific fragments, while 90.0% (279/310) of the InDel markers had polymorphisms among the 22 varieties. Most InDel markers detected 2 or 3 alleles, while some markers identified 6 genotypes (Fig. 2). Accordingly, 36 LInDel markers that were designed based on the LInDels between ZS97 and Nipponbare or between MH63 and Nipponbare were chosen to verify their availability (Additional file 7). One of them could not amplify any PCR products. The remaining 35 markers successfully amplified the PCR products, and 33 of them showed polymorphic amplicons (Additional file 8). In total, 312 of the verified LInDel markers (279 + 33) had polymorphisms in the 22 rice varieties.

### Polymorphism Information Content of the LInDel Markers

**Fig. 3:**
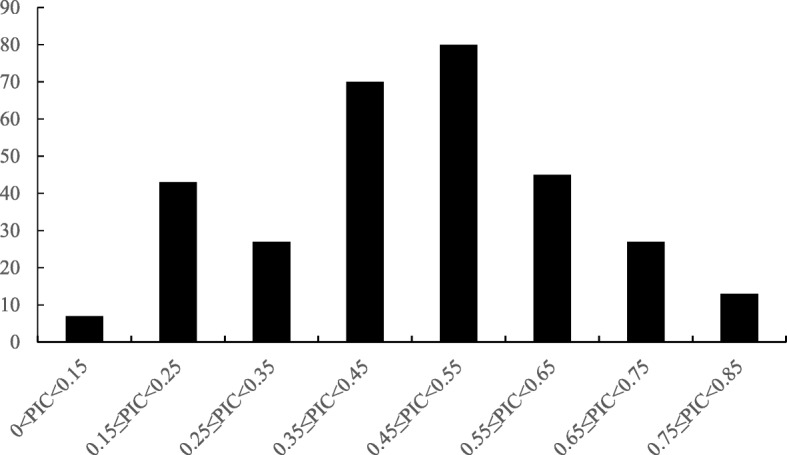
The PIC values of the 312 LInDel markers

The average PIC value of 279 LInDels was 0.45 with a range of 0.09 to 0.83 (Additional file 8). Most markers with a PIC value ranged from 0.35 to 0.55. Because these markers were designed based on *indica* rice references, the PIC values in the *indica* and *japonica* groups were calculated separately. The average PIC value in the *indica* group was as high as 0.45, while the PIC value in the *japonica* group was only 0.23. This result suggests that these markers were more polymorphic in the *indica* group than in the *japonica* group. In addition, the PIC values for the 33 *indica*/*japonica* LInDel markers were also calculated. The average PIC value was 0.53 with a range of 0.30 to 0.75. Following the same strategy mentioned above, the PIC values in the *indica* and *japonica* groups were calculated separately. There was no significant difference in the PIC values between the two groups. The average PIC value was 0.34/0.33 in the *indica* and *japonica* groups, and the range of the PIC value was 0–0.76/0–0.74 in the *indica* and *japonica* groups (Additional file 8). Finally, the PIC values of the 312 verified LInDel markers were calculated and used to draw a figure of the frequency distribution (Fig. 3).

### Cluster Analysis of the 22 Rice Varieties

A cluster analysis classified the 22 varieties into two main groups: group I and group II. Group I included 12 rice *japonica* varieties, such as Nipponbare, and Group II contained 10 *indica* varieties, including ZS97 and MH63 (Additional file 1: Figure S4). This classification was in agreement with the classification in the RiceVarMap database, except for the two varieties (Gangye and H360) that were not included in the RiceVarMap database (http://ricevarmap.ncpgr.cn) (Zhao et al. 2014). In group II, 6 of the rice varieties were *indica* varieties, and 3 varieties belonged to *Aus* rice, according to the RiceVarMap database. In fact, *Aus* rice belonged to *indica* rice (Xu et al. 2012). In summary, the 312 identified markers were well divided among the 22 varieties into the *indica* and *japonica* groups.

### LInDel Markers Located in or Closely Linked to Important Genes

**Fig. 4:**
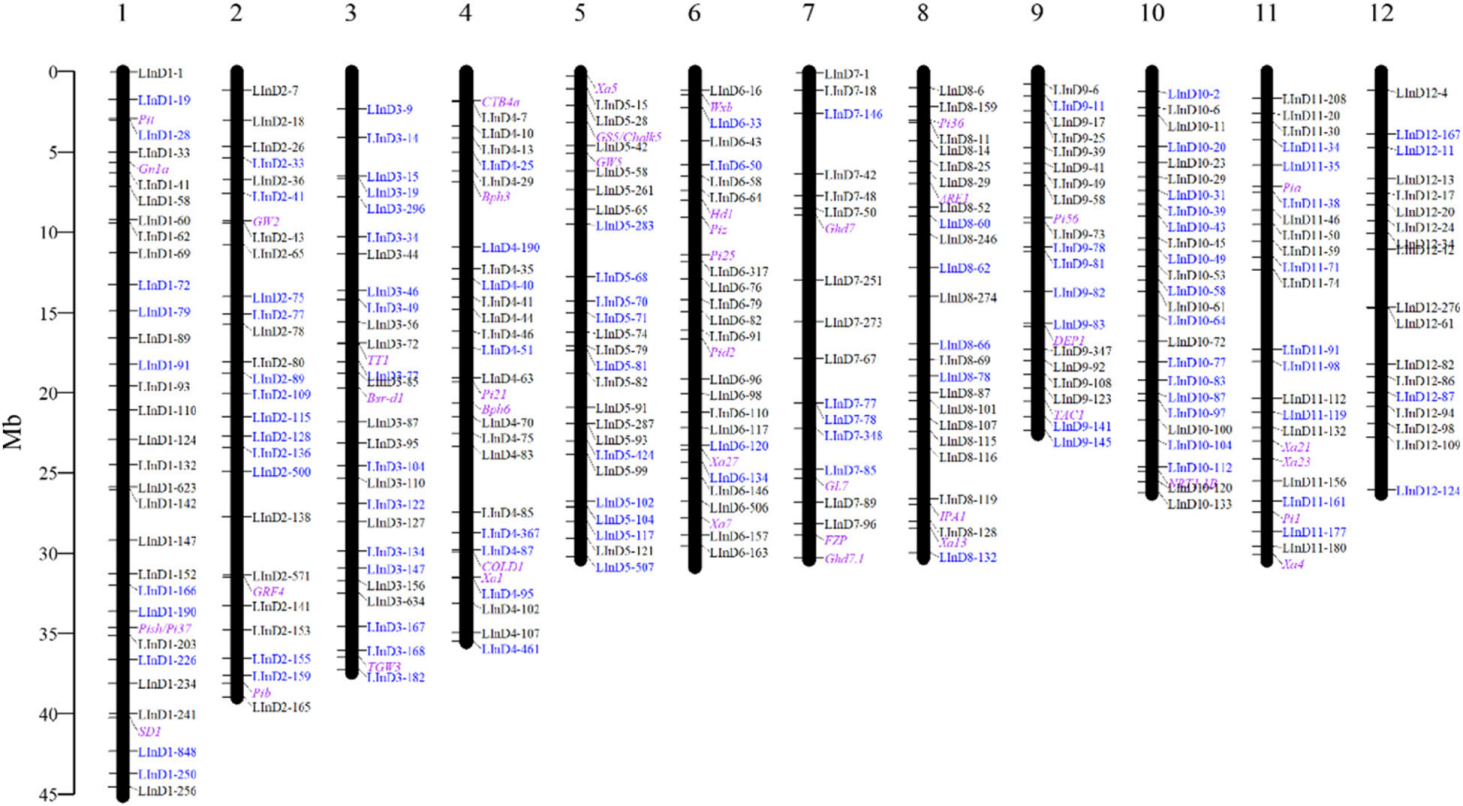
The physical map of the 55 breeding markers and the 312 LInDel markers. The breeding markers are labeled in purple, the markers with a PIC value larger than 0.5 are labeled in blue, and the markers with a PIC value smaller than 0.5 are labeled in black

Currently, hundreds of agronomically important genes have been cloned, such as *Ghd7*, *IPA1*, *GS3* and so on (Fan et al. 2009; Jiao et al. 2010; Miura et al. 2010; Xue et al. 2008). To develop markers for these important genes, we designed intragenic InDel markers for 55 genes based on the allele sequences of ZS97, MH63 and Nipponbare. Only 9 intragenic InDel markers were successfully designed (Additional file 9). The remaining 46 genes did not contain such a large size of InDels. Therefore, the most closely linked LInDel markers were chosen as alternatives (Fig. 4). We also listed the LInDel markers closely linked to the three following QTLs (gene not cloned): *Xa7* (Chen et al. 2008), *Bph15* (Lv et al. 2014) and *Bph25* (Yara et al. 2010). The nearest LInDel markers to reported markers GDSSR02 linked to *Xa7*, InD4 linked to *Bph15* and S00310 linked to *Bph25* were selected for potential marker assistant selection for target genes. We hereafter termed these 55 markers as breeding markers.

**Fig. 5:**
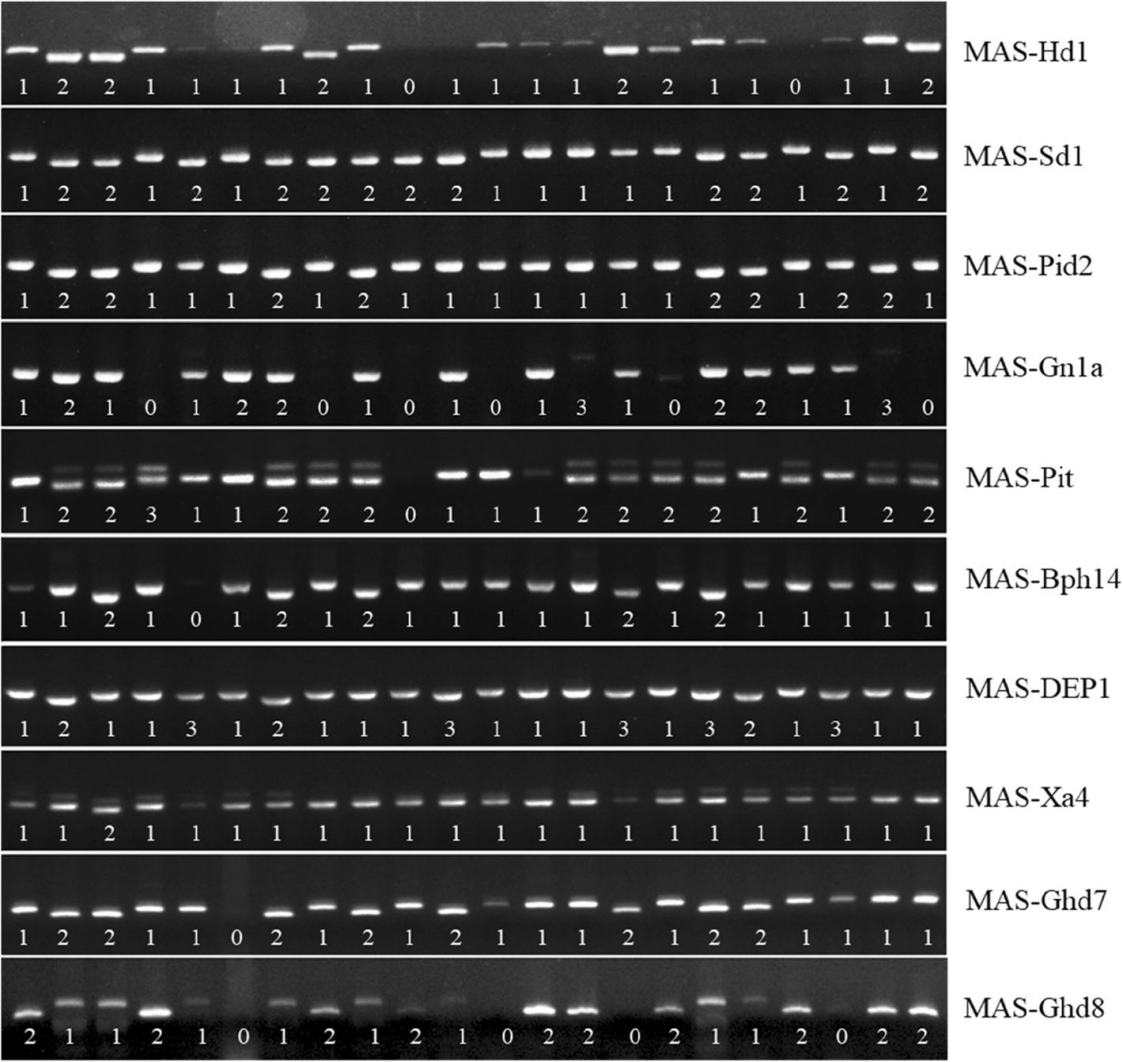
Genotypes of the 22 cultivars at 10 breeding marker loci. The samples from left to right are from the 22 rice varieties as follows: Zhonghua11, ZS97, MH63, Xizang2, N22, Nipponbare, 9311, 02428, Teqing, Taichung65, Chuan7, Haobuka, Wuyugeng, Gangye, H360, Zidao, Heimichut, Malaixizidao, Xiushui134, AUS449, Mitak and Cypress. The numbers under the bands represent different alleles

Ten breeding markers were chosen for experimental validation, including 6 intragenic markers derived from *Sd1*, *Pid2*, *Gn1a*, *Pit*, *DEP1* and *Xa4* and 4 markers closely linked to important genes (*Hd1*, *Bph14*, *Ghd7* and *Ghd8*). The 22 varieties were also used to test for polymorphism. Eight markers could amplify specific PCR products (Fig. 5). MAS-Hd1, MAS-SD1, MAS-Pid2, MAS-Pit, MAS-Bph14, MAS-DEP1, MAS-Ghd7 and MAS-Ghd8 had wide variation, with a PIC value ranging from 0.43 to 0.56. However, not all the DNA samples were amplified with MAS-Gn1a and MAS-Ghd8, most likely due to the variation of primer matching sites. The MAS-Xa4 had minimum variation, and only the MH63 genotype displayed a difference from the remaining 21 varieties.

### The Physical Map Consisted of 279 LInDel Markers and 55 Breeding Markers

These 312 verified LInDel markers and 55 breeding markers were placed into their corresponding positions in the physical map (Fig. 4). They were evenly distributed among all chromosomes, except a few large gaps on chromosomes 2, 4, 7, 8 and 12.

### Identification of Hybrids Using LInDel Markers

We generated 88 hybrid seeds between ZS97 and MH63 via artificial emasculation. To identify how many seeds are hybrids, two markers, LInD1–28 and LInD2–109, were used to genotype these seeds. An electrophoresis analysis of the InD1–28 PCR products in the 1.5% agarose gel showed that 7 hybrid seeds displayed the same band pattern as the female parent MH63, and 81 samples showed bands from both the male and female parents (Additional file 1: Figure S5A). Accordingly, an analysis of the LInD2–109 marker showed the same result as that with the LInD1–28 markers (Additional file 1: Figure S5B). Thus, the LInDel markers work stably and can be used for seed purity detection.

### Development of a Genetic Linkage Map with the LInDel Markers

To validate the feasibility of these LInDel markers to develop genetic linkage maps, 20 markers on chromosome 1 were chosen to genotype 96 F_2_ individuals that had been generated by self-pollinating F_1_ between ZS97 and MH63. A genetic linkage map covering the 171.0 cM chromosome region was constructed (Additional file 1: Figure S6). This genetic map demonstrated good collinearity with the physical position (Fig. 4). The average distance between the adjacent markers was 9.0 cM. In the distal section of the long arm, there was a large interval between LInD1–241 and LInD1–256 with a genetic distance of 33.5 cM, while the physical distance between these two markers was 4151.7 kb. In addition, four breeding markers had been successfully mapped to their corresponding positions.

## Discussion

### Universality of Agarose-Resolvable LInDel Markers in Rice

Currently, the reference genome sequences for five rice varieties have been released to the public, including sequences of the four following *indica* rice subspecies: 9311, ZS97, MH63, R498 (Du et al. 2017; Yu et al. 2002; Zhang et al. 2016a) and one *japonica* rice subspecies sequence of Nipponbare (Sasaki 2005). These released sequences made it possible to extract more SNPs and InDels among the different varieties, which provided an opportunity to design more DNA markers to improve future genetic studies and rice breeding strategies. ZS97 and MH63 are two representative *indica* rice varieties that belong to the *indica I* and *indica II* subpopulations (Xie et al. 2015). Therefore, the LInDel markers that are based on the reference genomes of these two varieties may be available between the *indica I* and *indica II* subpopulations. Nipponbare is a typical *japonica* rice variety, and the LInDel markers between ZS97 and Nipponbare and between MH63 and Nipponbare may represent the general polymorphisms that are present between the *indica* and *japonica* rice varieties. A total of 1838 and 4744 unique LInDel markers were developed between ZS97 and MH63 and between two different *indica varieties* and Nipponbare and had an average interval of 187.0 kb and 77.8 kb, respectively. A test of 279 LInDel markers in 22 varieties, including the *indica* and *japonica* varieties, showed that the polymorphisms among the genotypes were distinguishable with an agarose gel. The average PIC value in the 22 varieties was 0.45, and this indicated that the LInDel markers are universal across the rice varieties. However, the PIC values ranged from 0.09 to 0.83 for each LInDel marker site, indicating that there were some rare alleles at a few of the LInDel markers. The PIC values were 0.45 and 0.33 in 10 *indica* varieties and 12 *japonica* varieties, respectively. Hence, the number of polymorphic LInDel markers between any pair of varieties is not always enough in certain genomic regions. It is expected that more LInDels will be identified for marker development by comparing more reference genomes, after which, abundant LInDel markers should be obtained and the marker density would increase.

We chose 20 LInDel markers derived from ZS97 and MH63 to construct a genetic linkage map for chromosome 1 using an F_2_ population from the cross between ZS97 and MH63. The order of the positions of these markers in the linkage map was consistent with their physical order. In addition, consistent hybrid identification by different markers indicated that these LInDel markers worked well in an agarose gel. In addition, there are still many InDels of the 20–30 bp fragment that can be developed as InDel markers that use agarose gel (2.0%) electrophoresis for identification (Additional file 2); these markers will be used to develop saturated linkage maps.

### Alignment of Multiple Reference Sequences Enhanced the High Efficiency Development of LInDel Markers

The LInDel markers, including 279 from ZS97/MH63, 33 markers from *indica*/*japonica*, 10 breeding markers from *indica*/*japonica*, were validated and successfully amplified specific PCR products in the 22 rice varieties. To ensure specific amplification, all the primarily designed primers were examined by running an e-PCR program among the reference sequences in this study. Only the primers with unique amplicons were reserved for marker development. However, there are still some markers that could not amplify any PCR products in some rice varieties. For example, LInD3–104, LInD1–72 and LInD2–159 could amplify only the PCR products in 10–12 of the rice varieties (Additional file 8). The breeding markers MAS-Gn1a and MAS-Ghd8 could not amplify the PCR products of 4–5 of the rice varieties (Fig. 5). There are two main reasons for no amplification in some accessions: firstly, unlike the SSR marker, whose flanking sequences are conservative (Liu 2011; Tian et al. 2015), the flanking sequence of the LInDel markers may not be conservative enough, which may have been slight variations due to point mutations in the primer complementary regions in the 22 varieties, leading to a failed or inefficient match between the primers and the template DNA and resulting in failed amplification; secondly, the regions harbored some LInDel markers are entirely absent in some accessions, resulting in no amplification. To solve this problem, an alignment of multiple reference sequences will help to search for a conserved sequence that flanks the LInDels to develop primers. The database of multiple sequences alignments, such as TASUKE, will provide us a chance to optimize the primer binding sites and increase amplification efficiency of LInDel markers (https://ricegenomes.dna.affrc.go.jp/) (Kumagai et al. 2013).

### Agarose-Resolvable LInDel Markers for Breeders

Marker-assisted selection is necessary and can be performed with the help of DNA markers (Collard and Mackill 2007). The SSR markers that are resolved by both polyacrylamide gel electrophoresis and agarose gel electrophoresis are the most popular for MAS by breeders. Recently, High-Resolution Melting (HRM) (Liew et al. 2004; Wittwer et al. 2003) and Kompetitive Allele Specific PCR (KASP) (He et al. 2014; Semagn et al. 2014) were used for genotyping. Both HRM and KASP techniques have been used for high throughput genotyping as InDel makers (Steele et al. 2018; Zhou et al. 2015). In addition, the SNP array was another choice (Chen et al. 2014), which do not need electrophoresis either in agarose gel or in polyacrylamide gel. All three techniques (HRM, KASP, SNP array) do not require electrophoresis. But they need an expensive specific equipment for reading fluorescent dyes. That is why these techniques are not easy to be familiar with breeders in developing countries or even in China. These new technologies have not affected the use of SSR markers due to their low cost and high efficiency for large-scale genotyping (Kalia et al. 2011). However, agarose gel-based DNA markers are more welcome by breeders than above mentioned new technologies and PAGE-based SSR markers due to their ease of use and familiars to breeders. To promote MAS by breeders, we designed agarose-resolvable markers for 55 agronomically important genes, including 9 intragenic LInDel markers and 46 LInDel markers closely linked to these target genes. These markers are very useful tools for breeders. But it is noticed that the sizes of PCR products in Fig. 5 have no certain relationship with the target alleles for the important genes. It depends on the polymorphism between donor parental alleles and recipient parental alleles at target genes. If a large InDel variation existed between alleles of donor and receipt parents at target genes or closely linked regions, these LInDel markers would be utilized for marker aided selection in priority because they are intragenic markers or closely linked markers to the target genes. For example, to improve the resistance of a breeding lineage to brown plant hopper, the donor parent that carried the beneficial alleles of *Bph3* and *Bph14* was used to cross with the corresponding subspecies, and the *Bph3* intragenic marker LInD4–169 and the *Bph14*-linked marker LInD3–694 were used for tracking donor parent alleles in the following generations by running an agarose gel.

### Heteroduplex Formation of Large InDels in the Heterozygous Genotypes

**Fig. 6:**
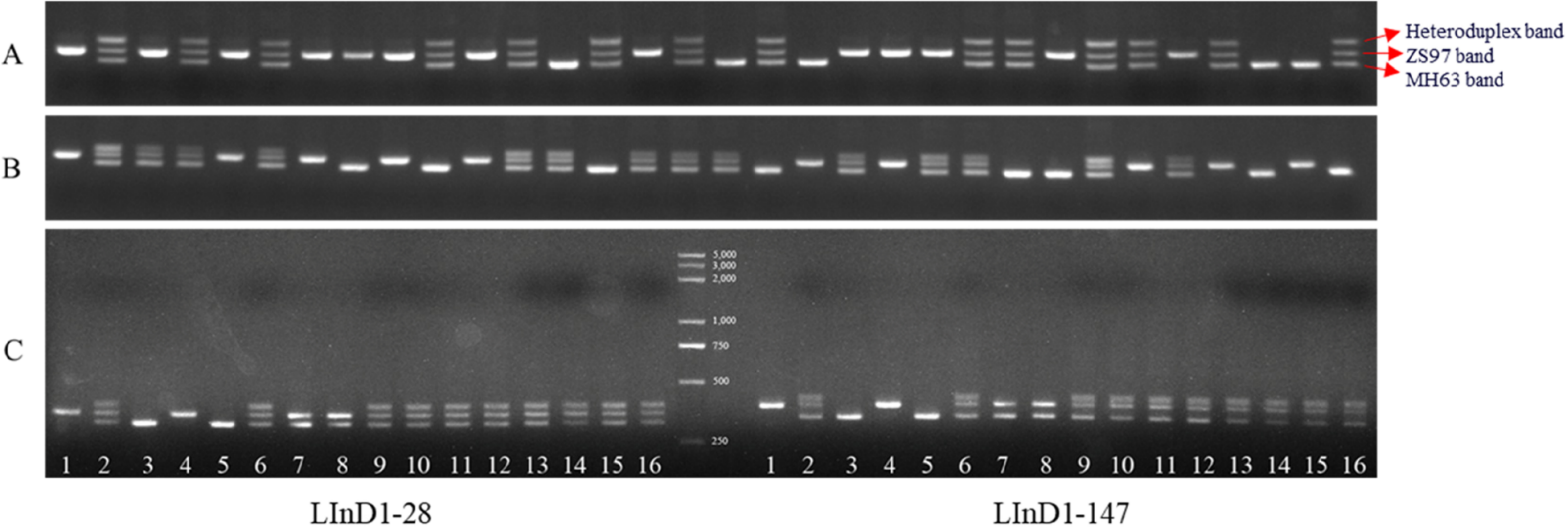
The genotypes of the F_2_ plants at the two LInDel marker loci and the verification of the heteroduplexes. **a** and **b** the genotypes of the F_2_ plants at the InDel marker loci of LInD1–28 and LInD1–147, three different bands were explained with red arrows. **c** the verification of the heteroduplexes. Lanes 1–10 are the nondenatured PCR products as follows: lanes 1–3, three F_2_ plants; lanes 4–6, ZS97, MH63 and their F_1_ cross SY63; lane 7, the mixed PCR products of the two types of homozygous F_2_ plants; lane 8, the mixed PCR products of ZS97 and MH63; lane 9, the PCR products amplified from the mixed DNA of two types of homozygous F_2_ plants; lane 10, the PCR products amplified from the mixed DNA of ZS97 and MH63; lanes 11–16, the denatured PCR products: lanes 11–12, the heterozygous F_2_ plant and SY63; lane 13, the mixed PCR products of the two types of homozygous F_2_ plants; lane 14, the mixed PCR products of ZS97 and MH63; lane 15, the PCR products amplified from the mixed DNA of the two types of homozygous F_2_ plants; and lane 16, the PCR products amplified from the mixed DNA of ZS97 and MH63

It is interesting that the PCR product of each cultivar was viewed as one band in agarose gels when we genotyped the 22 varieties using LInDel markers (Fig. 2). However, when we detected the genotype of the F_1_ or F_2_ individuals, we found that the heterozygotes displayed three bands (Additional file 1: Figure S5A, 5B and Fig. 6a, b), while the other two parents displayed only one unique band. It is possible that the three bands were the products of two homoduplexes and one heteroduplex, which was a double-stranded (duplex) molecule of nucleic acid that originated from the genetic recombination of single complementary strands between the insertion and deletion alleles. Thus, in this study, the heteroduplex shifted slowest on the gel. The three DNA molecules could be readily distinguished from one to another due to their considerable difference in length of 30–55 bp.

To verify this hypothesis, the PCR products of the two homozygotes from the F_2_ populations were mixed together, and the PCR products from the parents ZS97 and MH63 were mixed together as the parallel control. Then, these mixed products were separated into two parts: one product was denatured at 95 °C and the temperature was slowly dropped to 25 °C at a rate of − 4 °C per minute, while the other product was set as a blank control without any denaturation. The mixed products that had not been denatured showed only two clear bands (Fig. 6c, lane 7, 8). The mixed PCR products that were denatured showed three bands (Fig. 6c, lane 15, 16). This is because the mixed, denatured PCR products provided the chance that the insertion and deletion molecules formed heteroduplexes.

In summary, we developed thousands of LInDel markers and 55 important gene-related breeding markers that can potentially be resolved by running an agarose gel. These markers would be good tools for MAS, which would attract more breeders to use it.

## Supplementary information

**Additional file 1: Figure S1.** The distribution of the InDel (20-90 bp) on the whole genome. **Figure S2.** The frequency of the InDel between 20 and 90 bp. Figure S3**.** Comparation of potential LInDel markers among three genomes. **Figure S4.** Dendrogram of 22 rice varieties derived by UPGMA from 312 LInDel markers. **Figure S5.** Detection of the hybrids Shanyou 63 from ZS97 and MH63 (MH63 as female parent). **Figure S6.** The genetic linkage map of 20 LInDel markers on chromosome 1. Four breeding markers were labeled on the corresponding position.

**Additional file 2.** The 20-90 bp LInDels between ZS97  and MH63.

**Additional file 3.** The 30-55 bp LInDels between ZS97 and MH63 for markers design.

**Additional file 4.** The 1838 unique LInDel markers designed by 30-55 bp LInDels.

**Additional file 5.** The 30-55 bp LInDels between ZS97 and Nipponbare or between MH63 and Nipponbare.

**Additional file 6.** The 4744 unique LInDel markers designed by the 30-55 bp LInDels

**Additional file 7.** The 346 LInDel markers chosen for validation.

**Additional file 8.** The genotype of 22 rice varieties detected by 312 LInDel markers.

**Additional file 9.** The design of 55 breeding markers.

## Data Availability

The classification of the 22 rice cultivars was based on the RiceVarMap database (http://ricevarmap.ncpgr.cn/v2/). The genome sequences (the first release version) of ZS97 and MH63 were downloaded from the Rice Information Gateway database (http://rice.hzau.edu.cn/cgi-bin/rice/download_ext). The Nipponbare genome reference was downloaded from the Rice Genome Annotation Project database (http://rice.plantbiology.msu.edu/pub/data/Eukaryotic_Projects/o_sativa/annotation_dbs/pseudomolecules/version_7.0/). The 55 agronomically important genes were targeted from the China Rice Data Center (http://www.ricedata.cn/gene/). The sequences of the ZS97, MH63 and Nipponbare for these 55 genes were downloaded from the Rice Information Gateway database (http://rice.hzau.edu.cn/rice_rs1/) and the Rice Genome Annotation Project database (http://rice.plantbiology.msu.edu/).

## Abbreviations

HRM: High-Resolution Melting
InDel: Insertion-deletion
KASP: Kompetitive Allele Specific PCR
LInDels: Large fragment insertions/deletions
MAS: Marker-aided selection
MH63: Minghui 63
PCR: Polymerase chain reaction
PIC: Polymorphism information content
RAPD: Random amplified polymorphic DNAs
RFLP: Restriction fragment length polymorphism
SNP: Single nucleotide polymorphism
SSR: Simple sequence repeats
ZS97: Zhenshan 9
