# Supplementary material for: Development of Whole-Genome Agarose-Resolvable LInDel Markers in Rice
Source: Rice (N Y). 2020 Jan 6;13:1. doi: 10.1186/s12284-019-0361-3 (PMC6944724; doi:10.1186/s12284-019-0361-3)
Supplement: Supplementary file 1 — Additional file 1: Figure S1. The distribution of the InDel (20-90 bp) on the whole genome. Figure S2. The frequency of the InDel between 20 and 90 bp. Figure S3. Comparation of potential LInDel markers among three genomes. Figure S4. Dendrogram of 22 rice varieties derived by UPGMA from 312 LInDel markers. Figure S5. Detection of the hybrids Shanyou 63 from ZS97 and MH63 (MH63 as female parent). Figure S6. The genetic linkage map of 20 LInDel markers on chromosome 1. Four breeding markers were labeled on the corresponding position. [file 12284_2019_361_MOESM1_ESM.docx]

Supplementary Fig. 1 The distribution of the InDel (20-90bp) on the whole genome

Supplementary Fig. 2 The frequency of the InDel between 20-90bp


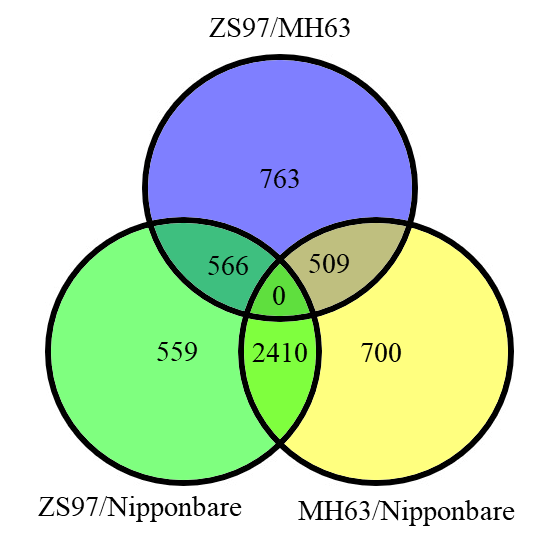


Supplementary Fig.3 Comparation of potential LInDel markers among three genomes


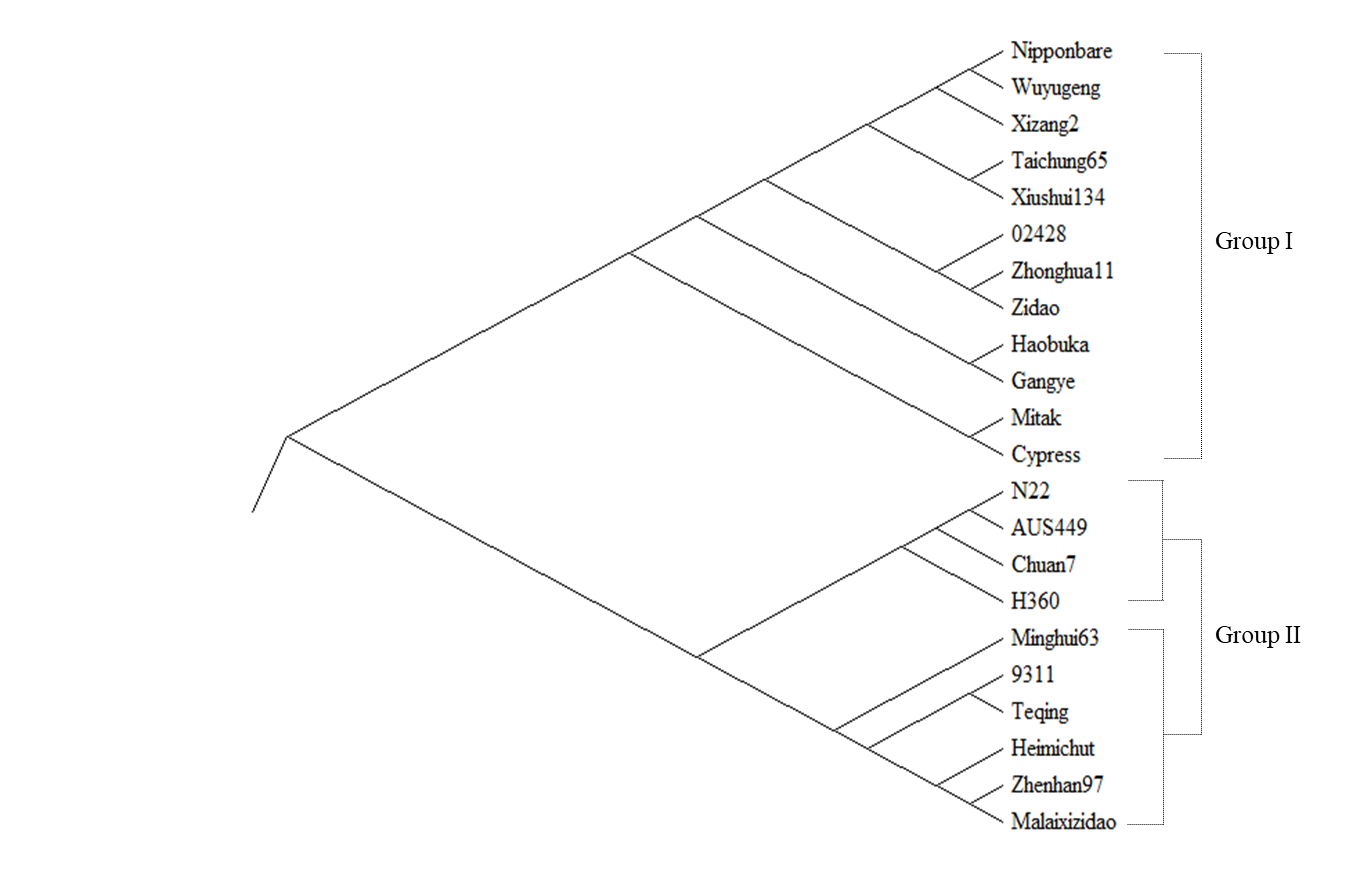


Supplementary Fig. 4 Dendrogram of 22 rice varieties derived by UPGMA from 312 LInDel markers


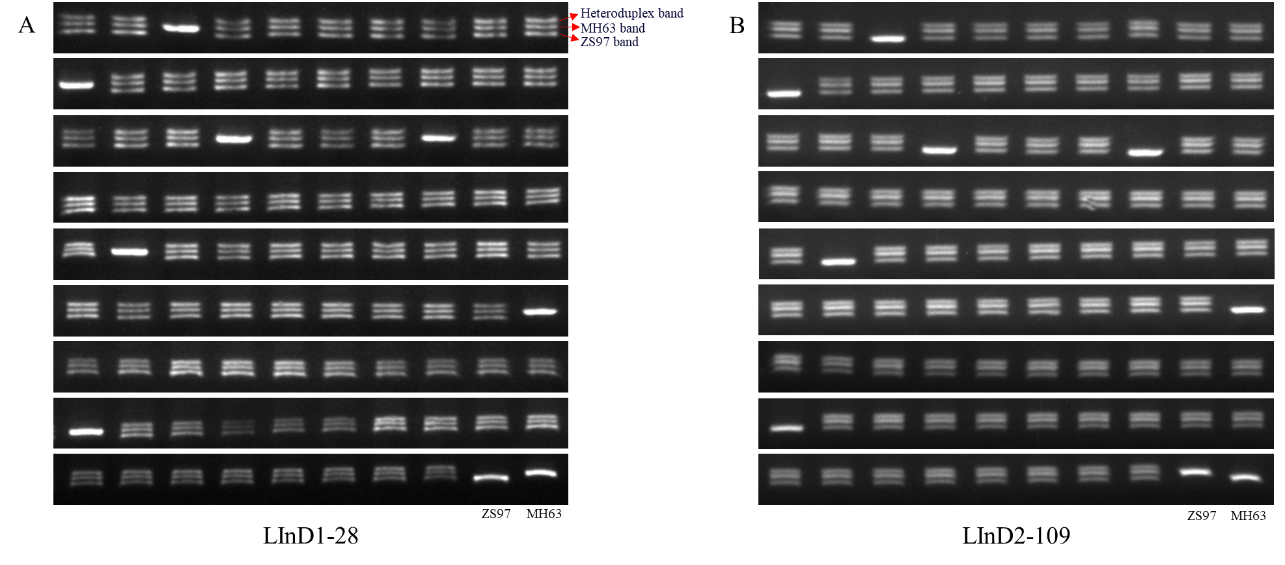


Supplementary Fig. 5 Detection of the hybrids Shanyou 63 from ZS97 and MH63 (MH63 as female parent)

90 samples, including 88 hybrid seeds and 2 parents. The last two samples were the parent ZS97 and MH63. Heterozygous genotype displayed three bands. Three different bands were explained with red arrows.


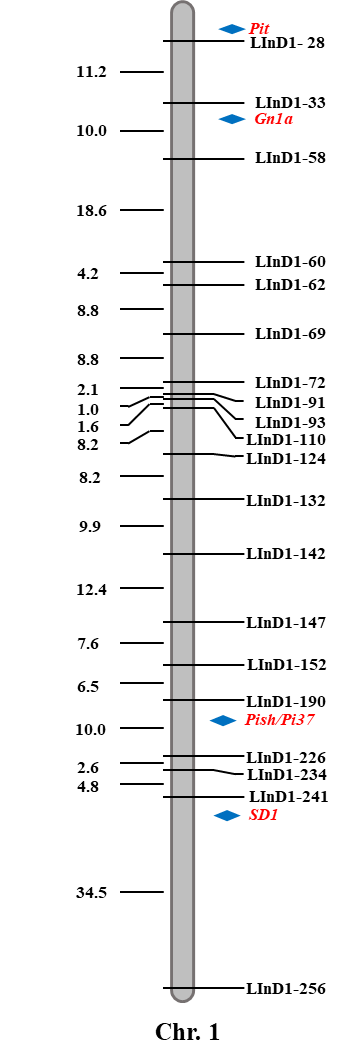


Supplementary Fig. 6 The genetic linkage map of 20 LInDel markers on chromosome 1. Four breeding markers were labeled on the corresponding position
